# Very Important Pool (VIP) genes – an application for microarray-based molecular signatures

**DOI:** 10.1186/1471-2105-9-S9-S9

**Published:** 2008-08-12

**Authors:** Zhenqiang Su, Huixiao Hong, Hong Fang, Leming Shi, Roger Perkins, Weida Tong

**Affiliations:** 1 Center for Toxicoinformatics, National Center for Toxicological Research (NCTR), U.S. Food and Drug Administration (FDA), 3900 NCTR Road, Jefferson, AR 72079, USA; 2 Z-Tech, an ICF International Company at FDA's National Center for Toxicological Research, Jefferson, AR 72079, USA

## Abstract

**Background:**

Advances in DNA microarray technology portend that molecular signatures from which microarray will eventually be used in clinical environments and personalized medicine. Derivation of biomarkers is a large step beyond hypothesis generation and imposes considerably more stringency for accuracy in identifying informative gene subsets to differentiate phenotypes. The inherent nature of microarray data, with fewer samples and replicates compared to the large number of genes, requires identifying informative genes prior to classifier construction. However, improving the ability to identify differentiating genes remains a challenge in bioinformatics.

**Results:**

A new hybrid gene selection approach was investigated and tested with nine publicly available microarray datasets. The new method identifies a Very Important Pool (VIP) of genes from the broad patterns of gene expression data. The method uses a bagging sampling principle, where the re-sampled arrays are used to identify the most informative genes. Frequency of selection is used in a repetitive process to identify the VIP genes. The putative informative genes are selected using two methods, t-statistic and discriminatory analysis. In the t-statistic, the informative genes are identified based on p-values. In the discriminatory analysis, disjoint Principal Component Analyses (PCAs) are conducted for each class of samples, and genes with high discrimination power (DP) are identified. The VIP gene selection approach was compared with the p-value ranking approach. The genes identified by the VIP method but not by the p-value ranking approach are also related to the disease investigated. More importantly, these genes are part of the pathways derived from the common genes shared by both the VIP and p-ranking methods. Moreover, the binary classifiers built from these genes are statistically equivalent to those built from the top 50 p-value ranked genes in distinguishing different types of samples.

**Conclusion:**

The VIP gene selection approach could identify additional subsets of informative genes that would not always be selected by the p-value ranking method. These genes are likely to be additional true positives since they are a part of pathways identified by the p-value ranking method and expected to be related to the relevant biology. Therefore, these additional genes derived from the VIP method potentially provide valuable biological insights.

## Background

DNA microarray technology [1,2] has rapidly advanced due to the intrinsic and unprecedented ability to simultaneously measure gene expression on a whole genome basis. Microarray technology continues to develop and is widely cited as offering much utility for translational science, from improved drug discovery, including target discovery, to improved clinical diagnostics and disease stage determination, prognostics and treatment selection, and more. With the prospect of microarray-derived biomarkers being applied in clinical applications, the bar is substantially raised for identification of informative genes enabling accurate classifiers, and efforts to this end are prevalent in the literature [3-11]. More specifically, there is a compelling need to identify a subset of genes from among the more than 20,000 in the entire genome that allow robust classifiers to be developed. The difficulty and challenge is to overcome the intrinsic characteristics of microarray data that contains a substantially small number of samples when compared to the number of genes [12,13]. These characteristics lead to the risk of fitting to noise as genes with high variability unrelated to phenotype masquerade as informative genes. The truly differentiating signals derived from small numbers of experimental replicates are difficult to distinguish in the sea of noise, leading to the appearance of unstable (i.e., non-reproducible) significant gene lists [14-16].

Gene selection is synonymous with feature selection or variable selection in machine learning, a process extensively used to mitigate the so called "curse of dimensionality" [17-20]. Generally, gene selection is done for either hypothesis testing or hypothesis generation. Selecting a subset of genes as molecular signatures or biomarkers that could be used for developing a generalized and accurate classifier for differentiating phenotypes is a hypothesis testing process [21], wherein rigorous validation is needed. On the other hand, identifying a list of putatively relevant genes related to a phenotype or endpoint of interest for subsequent research is a hypothesis generating process [22], wherein validation of the genes is much more relaxed; the genes so identified often shed light on the fundamental molecular mechanisms and biological processes under study.

Selecting and validating an "optimal" set of genes for a molecular signature or biomarker for a robust classifier is a complicated and time-consuming task. An exhaustive search encompassing all possible gene subsets to find the set yielding the smallest error can be an intractable computational task. Worse still, because the number of genes far outnumber samples, the potential for fitting to random noise is high, making stringent testing and validation essential [23,24].

Most methods to select informative genes for classification model development reported in the literature rely on ranked genes by fold change, correlation coefficient, or p-value from a t-statistic, Wilcoxon statistic, or analysis of variance (ANOVA), or some combination of these [22,25-30]. To a greater or lesser degree, all of these methods yield an informative gene list varying on the sample size, which has led doubt on microarray reliability [14-16]. In theory, true phenotype differentiating genes should be expected to express consistently with each other regardless of the sample size. In other words, the list of informative genes as well as the underlying mechanisms inferred by these genes should have nothing to do with the sample size.

In this study, a bagging [31] based new hybrid gene selection approach was investigated to identify informative genes. The rationale of the approach is that informative genes should consistently show significance for different variations of sample size. Accordingly, many re-sampling iterations are conducted to generate different variations of sample size and the frequency of genes exhibiting significance throughout the iterations formed the basis for identification of the informative genes that are considered as a Very Important Pool (VIP) of genes. In reality, the VIP genes can be identified using any existing gene selection approach or their combinations and can be used to derive molecular signatures to build robust classifiers with good generalization capability, or to narrow subsequent research to reveal relevant, fundamental molecular mechanisms in biological processes. In this study, t-statistic and discriminatory analysis are used to evaluate the significance of genes. In the t-statistic, the significant genes are identified based on p-values. In the discriminatory analysis, disjoint Principal Component Analyses (PCAs) are conducted for each class of samples, and those genes with high discrimination power (DP) [32] are identified as significant genes. The VIP genes are those having high frequency of showing significance in the re-sampling iterations. The utility of the proposed approach was demonstrated with nine diverse microarray datasets for identifying the informative genes for classifier development and compared with commonly used p-value ranking gene selection approaches.

## Results

**Figure 1:**
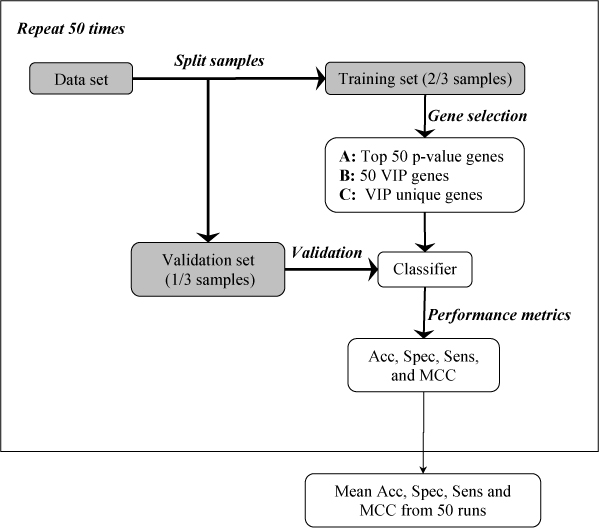
The flowchart for the classifier development and validation using three gene sets: (A) Top 50 p-value ranked genes; (B) Top 50 VIP genes; and (C) the unique VIP genes. Specifically, the data set is first randomly divided into two thirds of samples for training and the remainder for validation. Next, three sets of genes are generated solely based on the training set, and are subsequently used to develop Nearest-Centroid classifiers. Lastly, the classifiers are used to predict the validation samples and their respective prediction performance measured by accuracy (Acc), specificity (Spec), sensitivity (Sens), and Matthew's correlation coefficient (MCC) are calculated. The process is repeated 50 times and the averaged performance metrics are reported in Table 2.

The VIP gene selection approach for microarray based molecular signatures was applied to the nine publicly available microarray gene expression datasets described in Table 1. For the purpose of comparison, the p-value ranking method was also used. For each dataset, an unbiased sample splitting, gene selection, and validation dataset prediction process as depicted in Figure 1 was carried out. Briefly, a dataset is first randomly split into a training set with two thirds of the samples and a validation set with the remaining samples. With validation samples set aside, gene selection and classifier development are done using the training samples. Two lists of 50 genes are selected, one using the proposed VIP gene selection approach and the other using p-value ranking. The p-value ranking is based on an unpaired, two-tailed t-statistic with pooled variance estimate. In order to exam whether the VIP gene selection approach can identify informative genes or not, three sets of classifiers were generated, one for the VIP genes, one for the p-value genes and another for the genes uniquely identified by the VIP method (called unique genes hereafter). A Nearest-Centroid[33] classification method was used to develop classifiers. These classifiers are applied to predict the validation samples. The prediction performance of classifiers were compared by accuracies, specificities, sensitivities, and the Matthew's correlation coefficients (MCCs). The definitions of these measures are given in the section titled "materials and methods". The sample splitting, gene selection, and validation dataset prediction steps were repeated 50 times for adequate statistics.

**Table 1:** Nine microarray datasets used in the study.

**Name**	**Cancer type**	**Prediction task**	**Sample size**	**Number of events**	**Number of genes**	**Reference**
Beer	Lung adenocarcinoma	Survival	86	24	6532	[48]
Bhattacharjee	Lung adenocarcinoma	4-year survival	62	31	5403	[49]
Chen	Hepatocellular carcinoma	Tumors	156	82	3964	[50]
Pomeroy	Medulloblastoma	Medulloblastoma survival	60	21	7129	[52]
Rosenwald	Non-Hodgkin lymphoma	Survival	240	138	7399	[53]
Shipp	Diffuse large b-cell lymphoma (DLBCL)	Cured	58	32	6817	[54]
Singh	Prostate cancer	Tumors	102	52	12600	[55]
Yeoh	Acute lymphocytic leukaemia	Relapse-free survival	233	32	12236	[56]
van't Veer	Breast cancer	5-year metastasis-free survival	97	46	4948	[57]

We first compared the classifiers based on the VIP genes with those from the p-value ranking. As shown in Table 2, the VIP classifiers exhibited somewhat better performance compared to the classifiers from the p-value selected genes. The p-values from t-statistic for accuracy, specificity, sensitivity and MCC between two groups of classifiers (the VIP classifiers versus the p-value ranking classifiers) are 0.0027, 0.32, 0.059, and 0.0092, respectively. Therefore, at the 0.05 confidence level, the improvement of classifier measured in MCC and accuracy is significant, but not for specificity and sensitivity. The results indicate that the VIP genes may convey more, but not less, biologically relevant information than the p-value selected genes.

**Table 2:** Comparison of prediction performance for Nearest-Centroid classifiers built from unique VIP genes, top 50 p-value ranked genes, and 50 VIP genes. The classifier performance metrics, including accuracy (Acc), specificity (Spec), Sensitivity (Sens), and Matthew's correlation coefficient (MCC) were calculated based on averages of 50 repetitions of sample splitting, gene selection, and validation dataset prediction.

	**Unique VIP genes**					**50 p-value ranked genes**				**50 VIP genes**			
	
**Data set**	**Number of genes**	**Acc (%)**	**Spec (%)**	**Sens (%)**	**MCC**	**Acc (%)**	**Spec (%)**	**Sens (%)**	**MCC**	**Acc (%)**	**Spec (%)**	**Sens (%)**	**MCC**
Beer	15	64.7	38.3	74.0	0.13	64.7	38.3	74.0	0.12	65.2	35.4	75.6	0.11
Bhattacharjee	17	58.7	57.8	59.6	0.18	58.0	59.2	56.8	0.16	58.6	59.4	57.8	0.18
Chen	14	96.5	99.9	93.6	0.93	95.3	100.0	91.2	0.91	95.8	100.0	92.1	0.92
Pomeroy	20	60.8	54.0	64.2	0.19	60.8	51.7	65.3	0.18	62.4	55.7	65.8	0.22
Rosenwald	18	55.5	58.1	53.6	0.12	56.8	63.2	52.2	0.15	57.4	62.3	53.8	0.16
Shipp	18	51.6	50.8	52.5	0.03	47.9	51.8	43.0	-0.05	49.0	47.4	51.0	-0.02
Singh	15	94.3	98.3	91.7	0.89	98.1	100.0	96.9	0.96	97.8	100.0	96.4	0.96
Yeoh	22	74.6	37.8	80.2	0.15	78.2	31.0	85.4	0.15	80.2	35.0	87.0	0.21
van't Veer	20	64.8	64.8	64.9	0.30	65.2	61.5	68.6	0.31	66.9	66.1	67.6	0.34

Next, to determine whether the unique genes indeed contribute to the sample differentiation and thus biological relevance, we compared prediction performance of the classifiers built from unique genes with those built from the p-value ranked genes across the nine datasets. The average number of unique genes for each dataset is also listed in Table 2. It was shown that the average performance metrics (accuracy, specificity, sensitivity, and MCC) for classifiers built from unique genes (number from 14 to 22) are not very different from those built from top 50 p-value ranked genes for all nine datasets. The difference of each pair of average performance metrics is respectively tested across nine datasets with a null hypothesis that the compared performance metrics (accuracy, specificity, sensitivity, or MCC) is not very different from each other by using a paired and two-tailed t-statistic. The p-values given by t-statistic are 0.63, 0.77, 0.95, and 0.81 for accuracy, specificity, sensitivity, and MCC respectively. Apparently, the differences of all prediction performance metrics among classifiers are not significant at the 0.05 confidence level. This suggests that the unique VIP genes are statistically equivalent as those identified by p-value ranking in distinguishing different types of samples. Therefore, these unique genes could be an additional subset of genes which are equally as important as those selected with p-value ranking. The existence of additional subsets of classifying genes may imply that there exist multiple biological processes for studied endpoints or co-factors.

Lastly, to gain more understanding of the VIP genes in terms of biology related to the investigated dataset, we further examined the unique genes as well as the common genes shared by the p-value method in the van't Veer dataset using PathArt  through the FDA genomic tool, ArrayTrack . PathArt is a pathway analysis tool that contains disease related canonical pathways manually created from the literature. The van't Veer dataset contains 24 unique genes and 26 common genes. Of 24 unique genes, ten genes were found in PathArt and were listed in Table 3. Most of these ten genes involve biological processes related to various cancers; for example, IGFBP5 and MMP9 are directly related to breast cancer. We also examined the pathways associated with the 26 common genes and found seven unique genes were involved in seven pathways identified by the common genes (Table 4). These results demonstrate that the unique genes not identified by the p-value ranking could convey additional important information for biological interpretation.

**Table 3:** Pathways identified for the unique VIP genes and common genes for the van't Veer dataset.

	**Accession number (Symbol)**	**Full Name**	**Pathway name**	**Category (e.g. disease)**
**Unique VIP genes**	AF055033 (IGFBP5)	Insulin-like growth factor binding protein 5	Estrogen signaling pathway	Breast cancer
			IGF signaling pathway	Lung cancer
	NM_000599 (IGFBP5)		AR mediated pathway; insulin-like growth factor-1 signaling pathway	Prostate cancer
			Responsive genes	Ovarian cancer
	NM_000017 (ACADS)	Acyl-coenzyme A dehydrogenase, C-2 to C-3 short chain	Responsive genes	Colon cancer
	NM_004994 (MMP9)	Matrix metallopeptidase 9 (gelatinase B, 92 kDa gelatinase, 92 kDa type IV collagenase)	Heregulin, and CXCL12 signaling pathway	Breast cancer
			Bombesin, IL10, IL8, TGFbeta, and HGF signaling pathway; responsive genes	Prostate cancer
			Responsive genes; thrombospondin signaling pathway	Pancreatic cancer
			Gastrin, HGF, and IL4 signaling pathway; integrin, and UPAR mediated pathway	Colon cancer
			Responsive genes	Chronic myeloid leukemia
			EGF signaling pathway; VEGF mediated pathway; responsive genes	Ovarian cancer
			HGF, and IL6 signaling pathway; Responsive genes	Lung cancer
	NM_001197 (BIK)	BCL2-interacting killer (apoptosis-inducing)	p53 mediated pathway	Colon cancer
	NM_001809 (CENPA)	Centromere protein A	Responsive genes	Lung cancer
			p21 mediated pathway	Cell-cycle
	NM_002808 (PSMD2)	Proteasome (prosome, macropain) 26S subunit, non-ATPase, 2	Tat signaling pathway	Acquired immuno deficiency syndrome
	NM_004336 (BUB1)	BUB1 budding uninhibited by benzimidazoles 1 homolog (yeast)	Spindle Checkpoint Pathway	Cell-cycle
	NM_004626 (WNT11)	Wingless-type MMTV integration site family, member 11	Cell-cell signaling pathway	Others
			WNT receptor signaling pathway	Others
	NM_004887 (CXCL14)	Chemokine (C-X-C motif) ligand 14	Signal transduction pathway	Others

**Common genes**	AL050227 (PTGER3)	Prostaglandin E receptor 3 (subtype EP3)	Estrogen signaling pathway	Breast cancer
			PGE2 mediated pathway	Lung cancer
	NM_006763 (BTG2)	BTG family, member 2	Estrogen signaling pathway	Breast cancer
			Responsive genes	Prostate cancer
			CEBP alpha mediated pathway	Chronic myeloid leukemia
			Miscellaneous	DNA repair
			BTG mediated pathway	Cell-cycle
	NM_003862 (FGF18)	Fibroblast growth factor 18	WNT signaling pathway	Colon cancer
	NM_006115 (PRAME)	Preferentially expressed antigen in melanoma	Responsive genes	Ovarian cancer
	X05610 (COL4A2)	Collagen, type IV, alpha 2	Responsive genes	Glioblastoma
	NM_003981 (PRC1)	Protein regulator of cytokinesis 1	p21 mediated pathway	Cell-cycle
	NM_006027 (EXO1)	Exonuclease 1	p21 mediated pathway	Cell-cycle
	NM_002811 (PSMD7)	Proteasome (prosome, macropain) 26S subunit, non-ATPase, 7	Tat signaling pathway	Acquired immuno deficiency syndrome

**Table 4:** The pathways involved with both unique VIP genes and common genes for the van't Veer dataset

**Pathway name**	**Unique gene**	**Common gene**	**Category**
Estrogen signaling pathway	IGFBP5 (AF055033, NM_000599)	BTG2, PTGER3	Breast cancer
p21 mediated pathway	BUB1B, CENPA	EX01, PRC1	Cell-cycle
CEBPalpha mediated pathway	MMP9	BTG2	Chronic myeloid leukemia
WNT signaling pathway	WNT11	FGF18	Colon Cancer
Tat signaling pathway	PSMD2	PSMD7	Acquired immuno deficiency syndrome
Responsive genes	MMP9	BTG2	Prostate cancer
Responsive genes	MMP9, IGFBP5 (AF055033, NM_000599)	PRAME	Ovarian cancer

## Discussion

Quantitatively assessing the effectiveness of gene selection methods can be problematic owing to several limitations among which selection bias caused by information leakage from training phase to validation phase figures prominently [24]. The most severe bias was described by Ambroise *et al*. [21] and Simon *et al*. [34] as occurring when identifying genes from the entire dataset (i.e., training set and validation set) and using them in cross-validation. Wessels *et al*. [35] and Lai *et al*. [24] describe a less severe bias. Typically, the training samples are used to generate a series of gene subsets, while the performance of a classifier trained with the training samples and tested with the validation samples is applied to estimate the informativeness of each gene subset. The bias derives from the fact that the validation samples are used to select the best performing gene subset. Since optimization of the gene subset is part of the training process, selection of the best gene subset should be conducted with the training samples only. This process as shown in Figure 1 has been carried out in this study to assess the utility of the proposed VIP gene selection method by entirely avoiding bias due to information leakage from validation dataset in training phase.

Classification method selection is another important aspect of developing predictive models from microarray expression data. Many classifiers are created with one or more adjustable parameters that affect not only the prediction accuracy but also the complexity of the classifiers and the computational expense of their use. The proper adjustment of the tuneable parameters can affect the fairness of comparative predictive performance assessments. For example, the relatively simple k-Nearest Neighbour (KNN)[36] classification method has a tuneable k in the prediction rules. Adjusting k requires some validation process be carried out. Generally, different validation strategies such as leave-one-out cross validation, k-Fold cross-validation, or Monte Carlo validation, will yield different preferred values of k. Other classification approaches, such as Support Vector Machine (SVM) [37], Partial Least Squares Discriminant Analysis (PLS-DA) [38], Random Forest (RF)[22], and Artificial Neural Networks (ANN) [39] are considerably more complex by comparison, causing more work and computational cost. According to Wessels *et al*. [35], Michiels *et al*. [33], and Lai *et al*. [24], choosing a classification method with a limited complexity can help prevent over-training, thus providing a more robust predictor. In this study, the simple classification approach Nearest-Centroid was used to develop and compare classifiers based on unique VIP genes and top 50 p-value ranked genes. Since the method lacks a tuneable parameter, risks of overtraining are lessened compared to other methods, as are the chances that differences in prediction accuracy are due to method rather than selected genes.

Commonly used gene selection approaches in DNA microarray data analysis, such as p-value ranking or fold change ranking and others, assume that all genes are stochastic variables that are unrelated to each for purposes of calculating significance. This assumption is inconsistent with the actual biological processes where most genes have some interdependency to and are interlinked with other genes through complex mechanisms and pathways. In contrast, the proposed VIP gene selection approach uses both DPs and p-values to assess the discriminatory capability of genes in differentiating sample types. DPs are calculated from two independent PCAs that fuse discriminating information across whole genes. The interdependence and interlinking effects among genes are embedded within the DP calculation, enhancing rather than reducing many aspects of actual biological processes. Furthermore, the bagging re-sampling technique, which has been used to analyze microarray data for clustering [40-42] and classification [43-46], is used here to mitigate the chance selection of genes. Compared with p-value ranking-type gene selection approaches, the proposed VIP gene selection has great potential to select additional informative genes that can be useful for either biological insights or to improve the prediction performance of classifiers.

## Conclusion

The new hybrid gene selection approach was investigated for identifying VIP genes from nine diverse gene expression datasets. The VIP gene selection approach quantifies discriminatory capability for differentiating sample classes using both discrimination analysis and p-value ranking through a bagging sampling process. The classifiers built from those unique VIP genes showed comparable prediction capability to those built from the top 50 t-statistic based p-value ranked genes in predicting the types of unknown samples. Therefore, the VIP gene selection approach could provide an additional subset of genes which are of equivalent performance as those selected with the t-statistic based p-value ranking. The subset of VIP genes could convey additional biological information in terms of associated biological pathways and mechanisms during hypothesis generation. Similarly, the VIP genes could be used to improve molecular fingerprints for use in clinical biomarkers.

## Materials and methods

### Microarray datasets and software

Nine publicly available microarray datasets were used to demonstrate the relative prediction performance of the proposed VIP gene selection approach. The datasets are from Alon *et al*. [47], Beer *et al*. [48], Bhattacharjee *et al*. [49], Chen *et al*. [50], Gordon *et al*. [51], Pomeroy *et al*. [52], Resenwald *et al*. [53], Shipp *et al*. [54], Singh *et al*. [55], Yeoh *et al*. [56], and van't Veer *et al*. [57], that for convenience are hereafter respectively referred to as "Alon", "Beer", "Bhattacharjee", "Chen", "Gordon", "Pomeroy", "Resenwald", "Shipp", "Singh", "Yeoh", and "van't Veer"; information for each dataset is given in Table 1.

The VIP gene selection approach was developed using the programming language Matlab^® ^7.0, running on a DELL™ Precision 490 workstation equipped with two Intel^® ^Dual Core Xeon™ 3.0 GHz processors and 2 GB of memory. The Matlab codes are available upon request.

The biological interpretation of genes was conducted using PathArt  through the FDA genomic tool, ArrayTrack .

### Algorithm

**Figure 2:**
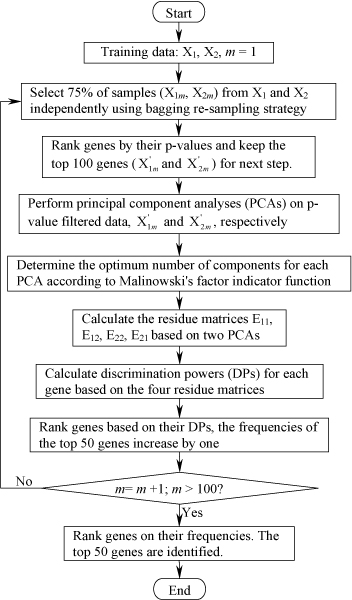
The detailed process for identifying a very important pool (VIP) of genes. X_1 _and X_2 _are, respectively, the gene expression profiles for class 1 samples and class 2 samples in the training set. X_1*m *_and X_2*m *_are samples randomly selected from X_1 _and X_2 _in the m^*th *^bagging step. ${\text{X}}_{1m}^{'}$ and ${\text{X}}_{2m}^{'}$ are the genes remaining after filtering genes from X_1*m *_and X_2*m*_, respectively. Malinowski's factor indicator function (IND) is calculated with equations $RE_{k}=\sqrt{\sum_{i=k+1}^{g}\lambda_{i}/p(n-k)}$ and *IND*_*k *_= *RE*_*k*_/(*n *- *k*)^2^, where *λ*_*i *_is the *i*^th ^eigenvalue of the total *g *eigenvalues; *n *is the number of samples and *p *is the number of genes. The optimum number (*k*) of components corresponds to the IND minimum. E_11 _and E_21 _are the residue matrices after projecting X_1*m *_and X_2*m *_into the PCA space for class 1, respectively, while E_22 _and E_12 _are the residue matrices after projecting X_2*m *_and X_1*m *_into the PCA space for class 2, respectively. The discrimination power (DP) of a gene *j *is calculated with the equation: $DP_{j}=[{\left(e_{j}^{12}\right)}^{\text{T}}(e_{j}^{12})+{\left(e_{j}^{21}\right)}^{\text{T}}(e_{j}^{21})]/\left[{\left(e_{j}^{11}\right)}^{\text{T}}(e_{j}^{11})+{\left(e_{j}^{22}\right)}^{\text{T}}(e_{j}^{22})\right]$, where $e_{j}^{11}$, $e_{j}^{12}$, $e_{j}^{22}$, and $e_{j}^{21}$ are the *j *columns of residue matrices E_11_, E_12_, E_22_, and E_21_, respectively.

The VIP gene selection approach combines discriminatory powers derived from two independent principal component analyses and p values from t-statistic to filter genes based on a bagging, re-sampling technique. The algorithmic process is depicted in Figure 2, where the training dataset is composed of *n*_1 _samples of class 1 and *n*_2 _samples of class 2. Samples of class 1 and class 2 are represented by the matrices **X**_1 _and **X**_2_, respectively. The VIP genes are chosen through the following steps:

1. Randomly select 75% of samples from the training data, **X**_1 _and **X**_2, _using a bagging, re-sampling strategy. The selected samples are represented with **X**_1*m *_for class 1 and **X**_2*m *_for class 2.

2. Rank genes by their p-values and only keep the top 100 genes for next step. P-values are calculated from a two-tailed and unpaired t-statistic with pooled variance estimate (i.e., equal variances or homoscedastic assumption) on **X**_1*m *_and **X**_2*m*_. The remaining data are represented by ${\text{X}}_{1m}^{'}$ and ${\text{X}}_{2m}^{'}$, respectively.

3. Rank genes based on their discrimination powers (DPs) and the increment the frequencies of the top 50 genes by one. The calculation of DPs is described in detail in the next section "calculation of discrimination power".

4. Repeat steps one through three 100 times.

5. Rank genes by frequencies and choose the top 50 genes as VIP genes.

### Calculation of discrimination power

DPs are calculated from two independent principal component analyses (PCAs). PCA is performed on each p-value-filtered data, ${\text{X}}_{1m}^{'}$ and ${\text{X}}_{2m}^{'}$ from step 2. The optimum number of components for each PCA is determined using Malinowski's factor indicator function (IND) [58] with eqs. (1) – (3):

(1) **X **= **TP**

(2) $$RE_{k}=\sqrt{\frac{\sum_{i=k+1}^{g}\lambda_{i}}{p(n-k)}}$$

(3) $$IND_{k}=\frac{RE_{k}}{{\left(n-k\right)}^{2}},$$

where **X **is either ${\text{X}}_{1m}^{'}$ and ${\text{X}}_{2m}^{'}$; **T **and **P **are the score and loading matrices of the PCA; *λ*_*i *_is the *i*^*th *^eigenvalue of the total *g *eigenvalues; and *n *and *p *are the number of samples and the number of genes in the matrix **X**, respectively. The optimum number (*k*) of components for the PCA is the one that yields the minimum *IND *value. The discrimination power (DP_*j*_) for a gene *j *can be calculated with eq. (4):

(4) $${\text{DP}}_{j}=\frac{{\left(e_{j}^{12}\right)}^{\text{T}}(e_{j}^{12})+{\left(e_{j}^{21}\right)}^{\text{T}}(e_{j}^{21})}{{\left(e_{j}^{11}\right)}^{\text{T}}(e_{j}^{11})+{\left(e_{j}^{22}\right)}^{\text{T}}(e_{j}^{22})},$$

where $e_{j}^{11}$, $e_{j}^{12}$, $e_{j}^{22}$, and $e_{j}^{21}$ are the *j *columns of matrices **E**_11_, **E**_12_, **E**_22_, and **E**_21_, respectively. **E**_11 _and **E**_12 _are the residue matrices after projecting ${\text{X}}_{1m}^{'}$ into the PCA spaces of class 1 and class 2, respectively, while **E**_22 _and **E**_21 _are the residue matrices after projecting ${\text{X}}_{2m}^{'}$ into the PCA spaces of class 1 and class 2, respectively. A residue matrix is calculated with eq. (5).

(5) **E **= **X **- **XPP**^T^,

where **E **is one of the four residue matrices **E**_11_, **E**_12_, **E**_22_, and **E**_21_.

### Prediction performance

The prediction performance of a Nearest-Centroid classifier in this study is characterized with four metrics: accuracy, specificity, sensitivity, and the Matthew's correlation coefficient (MCC). The metrics can be calculated from the prediction confusion matrix shown in Table 5 as follows:

**Table 5:** The prediction confusion matrix

**Observation**	**Prediction**	
	
	+1	-1
+1	TP (True positive)	FN (False negative)
-1	FP (False positive)	TN (True negative)

(6) $$Accuracy=\frac{TP+TN}{TP+TN+FP+FN}$$

(7) $$Specificity=\frac{TN}{TN+FP}$$

(8) $$Sensitivity=\frac{TP}{TP+FN}$$

(9) $$MCC=\frac{TP\times TN-FP\times FN}{\sqrt{\left(TP+FP)\times (TP+FN)\times (TN+FP)\times (TN+FN\right)}},$$

where TP, TN, FP, FN are respectively the numbers of true positive, true negative, false positive, and false negative predictions in the confusion matrix (Table 5).

## Disclaimer

The views presented in this article do not necessarily reflect those of the US Food and Drug Administration.

## Competing interests

The authors declare that they have no competing interests.

## Authors' contributions

ZS had the original idea, developed the method, did all calculations and data analysis, and wrote the first draft of manuscript. WT had the original idea, discussed on data analysis and presentation of results. HF, HH, LS, and RP involved in discussion on data analysis, verified some of the calculations and assisted with writing the manuscript. All authors read and approved the final manuscript.
